# Antidiabetic and Anti-Inflammatory Potential of Zingiberaceae Plants in Dietary Supplement Interventions

**DOI:** 10.3390/molecules31020311

**Published:** 2026-01-16

**Authors:** Natalia Kuzia, Olga Adamska, Natalia Ksepka, Agnieszka Wierzbicka, Artur Jóźwik

**Affiliations:** 1 Department of Biotechnology and Nutrigenomics, Institute of Genetics and Animal Biotechnology, Polish Academy of Sciences, Postępu 36a, Jastrzębiec, 05-552 Magdalenka, Poland; n.kuzia@igbzpan.pl (N.K.); n.ksepka@igbzpan.pl (N.K.); a.wierzbicka@igbzpan.pl (A.W.); 2 Department of Ophthalmology, Collegium Medicum, Cardinal Stefan Wyszyński University, Wóycickiego 1/3, 01-938 Warsaw, Poland; olgaadam98@gmail.com

**Keywords:** Zingiberaceae, antidiabetic, anti-inflammatory, ginger, curcumin, diabetes, inflammation, metabolic diseases, antioxidants, bioactive compounds

## Abstract

Plants from the Zingiberaceae family, particularly *Zingiber officinale*, *Curcuma longa*, and *Alpinia galanga*, are rich sources of bioactive compounds with documented antidiabetic and anti-inflammatory properties. This review summarizes current evidence on their phytochemical profiles and pathways relevant to metabolic regulation. Key compounds, including gingerols, shogaols, curcuminoids, and phenylpropanoids, support glucose homeostasis by enhancing insulin sensitivity, promoting Glucose Transporter Type 4 (GLUT4)-mediated glucose uptake, improving β-cell function, and modulating metabolic signaling pathways such as PI3K/Akt, AMPK, PPARγ, and NF-κB. Their potent antioxidant and anti-inflammatory activities further reduce oxidative stress and chronic low-grade inflammation, both central to the progression of type 2 diabetes and its complications. Evidence from selected clinical and experimental studies suggests that dietary supplementation with whole-rhizome preparations or standardized extracts (including formulation-enhanced products) may improve fasting blood glucose (FBG), glycated hemoglobin (HbA1c), lipid metabolism, and oxidative stress markers. Recent advances in delivery systems, including nanoemulsions, liposomes, and curcumin–piperine complexes, substantially enhance the bioavailability of poorly soluble phytochemicals, strengthening their therapeutic potential. Overall, Zingiberaceae plants emerge as promising natural supplements in nutritional and pharmacological strategies targeting diabetes. Further clinical research is required to refine dosage, confirm long-term efficacy, and support their integration into evidence-based metabolic interventions.

## 1. Introduction

Diabetes is one of the fastest-growing global health challenges of the 21st century. In 2024, an estimated 589 million adults (aged 20–79) were living with diabetes, and this number is projected to reach 853 million by 2050. Type 2 diabetes accounts for over 90% of all cases worldwide [1]. Diabetes mellitus (DM) is a multifactorial group of metabolic disorders characterized by chronic hyperglycemia resulting from impaired insulin secretion, insulin resistance, or both. The disease involves disturbances in carbohydrate metabolism, where glucose is simultaneously underutilized as an energy substrate and overproduced through excessive hepatic gluconeogenesis and glycogenolysis, leading to persistent elevation of blood glucose levels [2]. The pathogenesis of DM reflects a complex interplay between genetic predisposition, environmental influences, and metabolic stressors that disrupt glucose homeostasis and insulin signaling pathways. Under physiological conditions, insulin secreted by pancreatic β-cells promotes glucose uptake in skeletal muscle and adipose tissue via the insulin receptor substrate (IRS)/phosphoinositide 3-kinase (PI3K)/protein kinase B (Akt) pathway while inhibiting hepatic gluconeogenesis and glycogenolysis [3]. Dysregulation of these mechanisms leads to systemic alterations in carbohydrate, lipid, and protein metabolism, contributing to oxidative stress, inflammation, and mitochondrial dysfunction that underlie the chronic complications of diabetes [3,4].

Diagnosis of diabetes is based on laboratory criteria established by international and national guidelines, as shown in Table 1. For individuals with borderline results (fasting blood glucose 100–125 mg/dL or HbA1c 5.7–6.4%), an oral glucose tolerance test (OGTT) is recommended to detect impaired glucose tolerance or early diabetes [2,5].

Diabetes is classified as type 1 diabetes (T1DM), type 2 diabetes (T2DM), gestational diabetes mellitus (GDM) and specific types of diabetes due to other causes, e.g., monogenic diabetes syndromes (such as neonatal diabetes and maturity-onset diabetes of the young), diseases of the exocrine pancreas (such as cystic fibrosis and pancreatitis), and drug- or chemical-induced diabetes (such as with glucocorticoid use, in the treatment of HIV/AIDS, or after organ transplantation) [2]. The current classification does not distinguish between latent autoimmune diabetes in adults (LADA), which is considered a clinical subtype of type 1 diabetes [5].

Type 1 diabetes (T1DM) is a chronic disease caused by autoimmune destruction of pancreatic β-cells. The disease results from both genetic risk and environmental triggers that alter immune pathways. T1DM arises from the cell-mediated autoimmune destruction of insulin-producing pancreatic β-cells by CD4+ and CD8+ T-cells and macrophages. There are four different markers for this pancreatic β-cell destruction, namely: islet cell autoantibodies, autoantibodies to insulin, autoantibodies to glutamic acid decarboxylase (GAD65), and autoantibodies to the tyrosine phosphatases IA-2 and IA-2b [6]. The incidence of type 1 diabetes increases during childhood and peaks between the ages of 10 and 14 years [7].

T2DM is characterized by a nonautoimmune, progressive loss of β-cell insulin secretion, typically in the presence of insulin resistance [8]. T2DM is strongly associated with obesity, sedentary lifestyle, and genetic predisposition [6,9]. Its pathophysiology involves impaired insulin signaling in the liver, skeletal muscle, and adipose tissue, accompanied by chronic inflammation, oxidative stress, and ectopic lipid accumulation, which together drive metabolic dysfunction [8,10].

The differential diagnosis between type 1 and type 2 diabetes can be challenging, particularly in adolescents and adults with obesity, who could be misclassified as having type 2 diabetes and be treated with oral medications [7].

Gestational diabetes mellitus is one of the most common medical complications of pregnancy. It is defined as glucose intolerance with onset or first recognition during pregnancy. GDM arises from insufficient β-cell compensation for pregnancy-related insulin resistance. This defect is thought to resolve after pregnancy but becomes manifest in later life as an increased risk of diabetes [11].

Inadequate glycemic control significantly increases the risk of developing chronic diabetic complications, such as retinopathy leading to blindness, recurrent limb infections that may result in amputations, diabetic nephropathy, and a broad spectrum of cardiovascular diseases. In recent years, increasing attention has been directed toward the investigation of bioactive compounds derived from herbs and plant extracts as potential sources of safer and more effective antidiabetic therapies [12].

**Figure 1:**
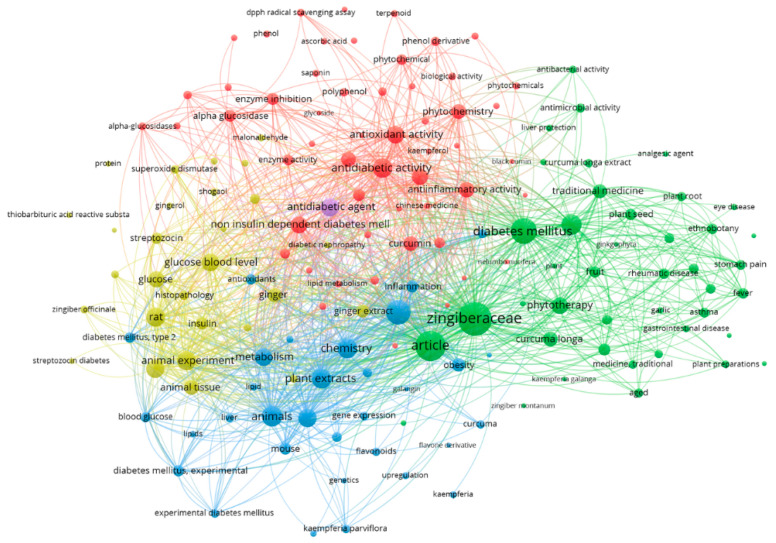
Co-occurrence map of key terms related to Zingiberaceae and diabetes, generated using VOSviewer 1.6.18 [17].

Due to the growing interest, bibliometric mapping highlights how research on Zingiberaceae has evolved within the broader field of diabetes-related phytotherapy. The resulting co-occurrence map (Figure 1) is divided into color-coded thematic clusters, each representing a distinct scientific direction. At the center of the network lies the term Zingiberaceae, exhibiting strong connections with numerous related concepts and emphasizing its central role in current research landscapes. 

The red cluster includes terms such as “antioxidant activity”, “enzyme inhibition”, “phenols”, “α-glucosidase”, “phytochemistry”, and “antidiabetic activity”. This cluster reflects intensive investigations into molecular actions of bioactive compounds, focusing on antioxidant effects, free radical scavenging, and inhibition of carbohydrate-digesting enzymes.

The green cluster embraces keywords linked to traditional medicine, such as “ethnobotany”, “phytotherapy”, “plant root”, “*Curcuma longa*”, and “plant seed”. It represents the long-standing integration of Zingiberaceae species in traditional medical systems and their application in preparations targeting diabetes, inflammation, and rheumatic disorders.

The yellow cluster highlights specific bioactive compounds and related terms such as “ginger”, “gingerol”, “shogaol”, “kaempferol”, “superoxide dismutase” and terms associated with lipid metabolism. This cluster focuses on gingerols, curcuminoids, and other metabolites involved in modulating oxidative stress and metabolic homeostasis.

The blue cluster captures experimental studies conducted in animal models and includes terms such as “rat”, “mouse”, “experimental diabetes mellitus”, “glucose blood level”, “insulin”, “liver”, and “gene expression”. This group reflects investigations into the effects of Zingiberaceae extracts on glycemia, insulin sensitivity, oxidative balance, and molecular pathways in vivo.

Beyond visual keyword clustering, Figure 1 highlights clear research trends and gaps. Earlier work is mainly linked to phytochemical characterization, whereas more recent research increasingly emphasizes molecular signaling pathways and experimental diabetes models. Notably, clinical and dietary supplement interventions studies remain scarce, particularly for *Alpinia galanga.*

Bibliometric data confirm that plants from the Zingiberaceae family are of great interest in relation to diabetes mellitus. The Zingiberaceae family consists of perennial herbs with creeping horizontal or tuberous rhizomes. It is comprised of approximately 52 genera and more than 1300 species that are distributed throughout tropical Africa, Asia, and the Americas. Many species are economically important as ornamental plants, spices, or are used in folk medicine [13]. These perennial, rhizomatous, and aromatic plants are recognized as one of the richest botanical sources of bioactive phytochemicals with well-documented therapeutic properties. Their rhizomes are characterized by a complex phytochemical composition, including phenolic compounds, terpenoids, alkaloids, and essential oils, which collectively underpin a wide spectrum of biological activities [14,15]. These components exert antidiabetic effects through multiple mechanisms. They act as antioxidants and anti-inflammatories, enhance glucose uptake via Glucose Transporter Type 4 (GLUT4), and preserve pancreatic β-cell function [16].

There has been growing interest in finding natural products from herbs and plants to control various diseases. While numerous studies investigating isolated phytochemicals are discussed to explain potential molecular mechanisms, this review emphasizes that the biological activity of Zingiberaceae species in dietary supplement interventions reflects the integrated action of multiple constituents within the complex phytochemical composition of the plant and cannot be directly interpreted from single-compound data.

The aim of this review is to present the phytochemical characteristics and antidiabetic mechanisms of selected Zingiberaceae species in the context of dietary supplement interventions in diabetes. In this review, the term “dietary supplement interventions” refers to targeted, dose-specific nutritional strategies implemented using preparations derived from the Zingiberaceae family with controlled composition and standardized content. These interventions include dried plant preparations, standardized extracts and preparations with increased bioavailability. We clearly distinguish such interventions from dietary intake, for which evidence from controlled clinical trials remains limited. Evidence from isolated phytochemicals is considered only to support the interpretation of relevant biological pathways and molecular targets and to underpin the principles of extract standardization.

## 2. Methodology

Interest in the relationship between the Zingiberaceae family and diabetes has increased noticeably over the past two decades. In Scopus, the term “diabetes” appears in more than 1.29 million documents, with over 860,000 published since 2010, reflecting sustained growth in research on its pathogenesis and treatment. The Zingiberaceae family is represented in 5379 publications overall, of which 4152 were published between 2010 and 2025.

A bibliometric analysis was conducted in Scopus, search date—March 2025, using the terms “Zingiberaceae” and “diabetes” in titles, abstracts and keywords. The search was restricted to original articles and reviews published between 2010 and 2025. Keyword co-occurrence mapping performed in VOSviewer 1.6.18 [17] was used to identify major thematic clusters related to antidiabetic mechanisms, traditional uses and experimental models.

This review follows a narrative, non-systematic approach and integrates preclinical and clinical findings on *Zingiber officinale*, *Curcuma longa*, and *Alpinia galanga* in the context of diabetes. Literature searches in PubMed and Scopus (2000–2025) included clinical trials, in vivo studies in diabetic or insulin-resistant models, and in vitro research focused on glucose metabolism, inflammation, and oxidative stress. Evidence was evaluated qualitatively, with attention to study design, dosing strategies and formulations. No formal risk-of-bias assessment or meta-analysis was performed.

## 3. Characteristics of Plants from the Zingiberaceae Family

Among the most notable representatives of this family are ginger (*Zingiber officinale* Roscoe), turmeric (*Curcuma longa* L.), Javanese ginger (*Curcuma xanthorrhiza* Roxb.), and galangal (*Alpinia galanga* L. Willd.) [15]. Z. *officinale*, *C. longa*, and *A. galanga* have attracted considerable scientific attention due to their rich phytochemical profiles and pharmacological potential. Extracts and active compounds obtained from these species exhibit multiple actions with antidiabetic relevance, reinforcing their long-standing use in traditional medicine and their emerging role in modern pharmacology [14].

### 3.1. Zingiber officinale Roscoe (Ginger)

*Zingiber officinale* Roscoe has a long history of culinary and medicinal use. Its rhizome has been used to alleviate ailments such as colds, nausea, vomiting, arthritis, and digestive discomfort, as well as for its antioxidant properties [18]. In addition to these traditional uses, ginger exhibits a wide range of pharmacological activities, including radioprotective, hepatoprotective, nephroprotective, neuroprotective, and gastroprotective effects, which have been extensively investigated at the molecular level [19,20]. The biological activity of *Z. officinale* comes from the content of volatile and non-volatile compounds. The volatile components are essential oils with a distinctive aroma, in contrast, the non-volatile components give a pungent, spicy taste, as summarized in Table 2 [21].

Volatile compounds, representing about 2–3% of the rhizome, constitute the essential oil fraction and include sesquiterpenes such as zingiberene, curcumen, farnesene, zerumbone, β-elemene, β-sesquiphellandrene, and monoterpenes such as cineol, linalool, borneol, citral, limonene and α/β-pinene. Additional terpenes such as β-bisabolene, α-curcumene, and α-farnesene are considered major constituents of ginger essential oil [22,23].

Non-volatile compounds are largely responsible for the pungency and pharmacological activity of ginger. The key phenolic compounds include gingerols, shogaols, paradols, and zingerone. In fresh rhizomes, 6-, 8-, and 10-gingerol predominate, while heat treatment or storage causes dehydration to form shogaols, which, upon hydrogenation, yield paradols. Zingerone, absent in fresh rhizomes, forms during drying or roasting through retro-aldol reactions of gingerols [23].

*Zingiber officinale* also contains other phenolics such as quercetin, gingerenone-A, and 6-dehydrogingerdione, as well as polysaccharides, lipids, organic acids, and crude fibers contributing to its nutritional profile. The concentrations of these compounds depend on the plant variety, cultivation conditions, and processing methods, which can affect their bioactivity and therapeutic potential [23,24,25].

**Table 2:** Major bioactive constituents and pharmacological activities of *Zingiber officinale* Roscoe.

Plant	Fraction	Chemical Type	Examples of Main Compounds	Effect	Reference
*Zingiber officinale* Roscoe	Volatile	Monoterpenes	Camphor, camphene, limonene, neral, geranial	Anti-inflammatory, antibacterial, antioxidant, digestive, cardioprotective, antispasmodic	[15,26,27,28]
		Sesquiterpenes	β-bisabolene, α-zingiberene, β-sesquiphellandrene, ar-curcumene, farnesene, zerumbone		
	Non-volatile	Phenols	6-gingerol, 8-gingerol, 6-shogaol, zingerone	Antioxidant, anti-inflammatory, anticancer, antidiabetic, antiallergic, antiemetic, lipid-lowering, heart-protective, supporting glucose metabolism, anti-obesity	[15,26,27,28,29]

### 3.2. Curcuma longa L. (Turmeric)

*Curcuma longa* L. is a perennial herb native to India and widely cultivated across Asia. It has been used in traditional systems of medicine such as Ayurveda for centuries due to its culinary, nutritional, and therapeutic properties [30]. The non-volatile fraction of *C. longa* is dominated by curcuminoids, the polyphenolic pigments responsible for its characteristic yellow color. Commercial curcumin mixtures typically contain approximately 80% curcumin (CUR), 15% demethoxycurcumin (DMC), and 3% bisdemethoxycurcumin (BDMC). High-performance liquid chromatography (HPLC) analyses report curcumin concentrations of about 3.2 mg/g dry extract, DMC 2.3 mg/g, and BDMC 0.25 mg/g [30,31].

Curcumin is a lipophilic and poorly water-soluble compound but remains stable in acidic environment. Despite strong antioxidant potential, it shows poor bioavailability due to rapid metabolism and low absorption [32]. *C. longa* also contains a wide range of phenolic compounds identified by HPLC, including caffeic acid, coumaric acid, quercetin-3-D-galactoside, and others [31].

The volatile fraction of *C. longa* consists of essential oils accounting for 3–7% of the dry rhizome weight, dominated by sesquiterpenes such as ar-turmerone (up to 40%), α-turmerone (10–25%), and curlone (~23%), which contribute to both aroma and bioactivity [33].

These volatile and non-volatile compounds shape the broad therapeutic potential of *C. longa*, as summarized in Table 3. Curcuminoids and other non-volatile phenolic compounds primarily function by enhancing antioxidant defenses and suppressing inflammatory signaling, while volatile sesquiterpenes such as ar-turmerone complement these actions through antimicrobial and cytoprotective effects. *C. longa* influences oxidative balance, inflammation, and cellular homeostasis, supporting its long-standing use in traditional and modern medicine [34,35].

### 3.3. Alpinia galanga (Greater Galangal)

*Alpinia galanga* L. Willd., commonly known as greater galangal, is a perennial herbaceous plant belonging to the Zingiberaceae family, widely used as both a culinary spice and a medicinal herb in Ayurveda, Traditional Chinese Medicine, and Thai folk medicine [38]. It grows in humid tropical regions across Southeast Asia, including Indonesia, India, Malaysia, and Thailand [39]. Its rhizomes are rich in phenylpropanoids (e.g., 1′S-1′-acetoxychavicol acetate (ACA) and 1′S-1′-acetoxy-eugenol acetate (AEA)), flavonoids (e.g., galangin, pinocembrin, apigenin), phenolic acids, and essential oils [40,41].

The phytochemical composition of *A. galanga* comprises both volatile and non-volatile metabolites. The volatile fraction, forming approximately 0.5–1.5% of the rhizome depending on variety and processing, is rich in monoterpenes and sesquiterpenes. Gas chromatography-mass spectrometry (GC-MS) analyses consistently identify 1,8-cineole as the dominant component, accompanied by α-fenchyl acetate, β-myrcene, β-ocimene, camphor, limonene, and other terpenoids [40]. These compounds largely account for its aromatic profile as well as antimicrobial and anti-inflammatory properties. The non-volatile fraction includes phenylpropanoids such as ACA and AEA, flavonoids including galangin, pinocembrin, and apigenin, and various phenolic acids [41].

*A. galanga* exhibits a broad pharmacological spectrum, including antidiabetic, antioxidant, anticancer, antimicrobial, hepatoprotective, and gastroprotective effects (Table 4). These activities are mediated by attenuation of oxidative stress, inhibition of inflammatory cascades such as nuclear factor kappa-light-chain-enhancer of activated B cells (NF-κB), modulation of apoptotic pathways, and reported activation of metabolic regulators such as AMP-activated protein kinase (AMPK) and PI3K/Akt, contributing to improved glucose homeostasis and reduced insulin resistance [42,43].

The diverse phytochemical profile of *A. galanga*, encompassing metabolically active volatile and non-volatile constituents, underscores its value as a representative species of the Zingiberaceae family and a promising source of plant-derived therapeutic agents in metabolic and inflammatory disorders [40,43].

**Table 4:** Major bioactive constituents and pharmacological activities of *Alpinia galanga*.

Plant	Fraction	Chemical Type	Examples of Main Compounds	Effect	Reference
*Alpinia galanga*	Volatile	Monoterpenes	1,8-cineol, α-terpineol, β-pinene	Antibacterial, antifungal, anti-inflammatory, antioxidant, gastroprotective, anticancer	[43,44,45]
		Sesquiterpenes	Farnesene, germacrene D, β-caryophyllene		
	Non-volatile	Flavonoids	Galangin, quercetin	Antidiabetic, antioxidant	[44,46,47,48]
		Stilbenoids/ lignans	Pinostilbene, pinoresinol	Antioxidant, anti-inflammatory, immunomodulatory, antidiabetic, anticancer	[43,44,45]

## 4. Signaling Pathways and Molecular Mechanisms

Insulin is a β-cell-derived polypeptide hormone belonging to the same family as IGF-I/II and relaxin, and its secretion is primarily triggered by rising blood glucose and amino acid levels. Peripherally produced insulin also crosses the blood–brain barrier to act in the central nervous system (CNS) [49,50]. It is the master regulator of energy metabolism. It promotes glucose uptake and glycogen storage in the liver, muscle, and adipose tissue. It also stimulates lipogenesis and protein synthesis, while suppressing proteolysis and α-cell glucagon release [51,52]. Systemically, insulin suppresses hepatic glucose output, enhances peripheral glucose uptake, inhibits lipolysis, and signals mainly through the PI3K/Akt and mitogen-activated protein kinase (MAPK) pathways [10,53].

Because insulin is a central regulator of glucose homeostasis, molecules that influence its secretion, sensitivity, or signaling pathways have emerged as major focal points in contemporary diabetes research. The Zingiberaceae family is rich in bioactive compounds with promising antidiabetic properties. These plants have been widely used in traditional medicine, and recent research has explored their mechanisms, efficacy, and clinical relevance for diabetes management [54].

### 4.1. Zingiber officinale Roscoe (Ginger)

Bioactive compounds found in *Zingiber officinale*, particularly gingerols, shogaols, and paradols, exert pleiotropic effects on glucose homeostasis, pancreatic β-cell function, and inflammatory pathways. Clinical studies and meta-analyses show that ginger supplementation improves insulin sensitivity, enhances insulin secretion, and lowers fasting blood glucose (FBG) and HbA1c in patients with type 2 diabetes mellitus [55,56,57,58].

In randomized controlled trials, ginger intake was associated with improvements in Homeostatic Model Assessment for Insulin Resistance (HOMA-IR) and Quantitative Insulin Sensitivity Check Index (QUICKI) indices, as well as reductions in serum urea, although not all studies found significant changes in FBG and HbA1c [59]. Animal models demonstrate that ginger promotes regeneration of pancreatic islets by upregulating transcription factors Neurog3, Mafb, and Ins2, leading to restoration of β-cell mass and increased insulin secretion [60].

At the cellular level, *Z. officinale* enhances GLUT4 translocation to the plasma membrane of skeletal muscle and adipocytes, facilitating glucose uptake. It also inhibits α-amylase and α-glucosidase, delaying carbohydrate digestion, reducing postprandial hyperglycemia, and limiting glycation processes [61]. It has also been suggested that ginger exerts beneficial effects on lipid metabolism, including reductions in total cholesterol and triglycerides [55,58]. Bioactive compounds such as gingerols act as modulators of insulin signaling and activators of peroxisome proliferator-activated receptor gamma (PPARγ), improving insulin sensitivity and regulating adipogenesis [62,63].

Phenolic compounds like [6]-gingerol and [6]-shogaol inhibit tumor necrosis factor α (TNF-α), interleukin-6 (IL-6), and interleukin-1β (IL-1β), reduce nitric oxide production in lipopolysaccharide-stimulated macrophages, and suppress immune cell activation [61,63]. These compounds inhibit proinflammatory enzymes, including lipoxygenase and selected proteases. They decrease monocyte chemoattractant protein-1 (MCP-1), regulated on activation, normal T-cell expressed and secreted (RANTES), and myeloperoxidase activity, and enhance antioxidant defenses such as superoxide dismutase (SOD), catalase (CAT), and total antioxidant capacity (TAC) [25,57,64].

Natural antioxidants present in ginger, including tannins, flavonoids, and vitamins C and E, may help preserve β-cell viability and reduce blood glucose levels in a dose- and duration-dependent manner [65,66].

### 4.2. Curcuma longa L. (Turmeric)

*Curcuma longa* and its main polyphenol, curcumin, have multifaceted effects supporting glycemic control and improving pancreatic β-cell function. Randomized clinical trials have shown that curcumin supplementation improves β-cell function, reduces insulin resistance, and lowers fasting blood glucose and HbA1c [67,68]. Meta-analyses have also confirmed stabilization of HbA1c and reduction in progression from prediabetes to T2DM [69,70]. These mechanisms include activation of the AMPK pathway, increased translocation of GLUT4 transporters in skeletal muscle and adipose tissue, modulation of the PPARγ receptor, and regulation of adipokines such as adiponectin and leptin, which together promote glucose homeostasis [71].

Chronic low-grade inflammation plays a crucial role in the pathogenesis of insulin resistance and diabetic complications. *Curcuma longa* extracts inhibit the activity of key inflammatory pathways, including NF-κB, MAPK, toll-like receptor 4 (TLR4), nucleotide-binding Leucine-rich repeat and Pyrin domain containing 3 (NLRP3), and Janus Kinase/Signal Transducer and Activator of Transcription (JAK/STAT), by limiting the production of proinflammatory cytokines like TNF-α, IL-1β, IL-6, and inducible nitric oxide synthase (iNOS) [72,73]. In clinical studies, curcumin supplementation reduced C-reactive protein (CRP) and other inflammatory markers, confirming its pronounced anti-inflammatory effects in patients with type 2 diabetes. Oxidative stress is another key pathogenetic factor in the development of diabetes and its vascular complications. Curcumin acts both directly as a reactive oxygen species (ROS) scavenger and indirectly by increasing the activity of antioxidant enzymes such as SOD, CAT, and glutathione peroxidase (GPx) [69]. It also lowers the levels of markers of lipid peroxidation, such as malondialdehyde (MDA) and advanced glycation end products (AGEs), which protect endothelial cells and reduce the risk of developing diabetic nephropathy, retinopathy, and neuropathy [73,74].

Curcumin also exerts beneficial effects on glucose and lipid metabolism. Inhibiting the activity of digestive enzymes such as α-amylase and α-glucosidase limits glucose absorption in the intestine, reducing postprandial hyperglycemia [75,76]. Additionally, curcumin improves lipid profiles by lowering triglyceride, total cholesterol, low-density lipoprotein cholesterol (LDL-C), and very low-density lipoprotein cholesterol (VLDL-C) levels, while simultaneously increasing high-density lipoprotein cholesterol (HDL-C) levels [68,71]. These mechanisms are related to the inhibition of hepatic lipogenesis, increased fatty acid oxidation, and regulation of LDL receptor expression [69].

### 4.3. Alpinia galanga (Greater Galangal)

The hypoglycemic activity of *A. galanga* has been linked to phenylpropanoids such as (1′S)-1′-acetoxyeugenol acetate, which enhance glucose-dependent insulin secretion (GSIS) in β-cells by activating the IRS-2/PI3K/Akt pathway and the PDX-1 transcriptional controller, which regulates insulin gene expression. (1′S)-1′-acetoxyeugenol acetate inhibits α-glucosidase activity, reducing glucose absorption and further supporting glycemic control [77]. Flavonoids such as galangin, improve insulin-dependent function by translocating GLUT4 transporters and modulating PI3K/Akt distribution, which helps reduce insulin resistance [78].

Hydroalcoholic extracts from *A. galanga* rhizome exert potent anti-inflammatory effects by inhibiting the TLR4/MyD88/p38MAPK and JAK/STAT pathways in lipopolysaccharide-treated RAW 264.7 macrophages. This results in the release of proinflammatory cytokines (TNF-α, IL-6), nitric oxide (NO), and ROS, as well as the release of the anti-inflammatory cytokine IL-10. Importantly, the extract reduces the expression of inflammatory enzymes such as iNOS, cyclooxygenase-2 (COX-2), and matrix metalloproteinase-9 (MMP-9), and inhibits NF-κB nuclear translocation, confirming its immunomodulatory effects [79].

*A. galanga* flavonoids play a key role in neutralizing oxidative stress. Galangin limits lipid peroxidation increases the activity of antioxidant enzymes like SOD, CAT, GPx, and inhibits the activation of the NLRP3 inflammasome and NF-κB, reducing the inflammatory cascade [80,81].

In addition to its effects on insulin and glycemia, compounds from *A. galanga* contribute to the regulation of lipid metabolism. Preclinical studies have shown that galangin modulates the expression of PPARγ and glycogen synthase kinase-3 beta (GSK-3β) kinase, which contributes to improved lipid profiles and reduced triglyceride accumulation [81]. *Alpinia galanga* also contains phenolic compounds that act as α-amylase and α-glucosidase inhibitors, limiting starch hydrolysis and glucose absorption, thereby attenuating postprandial hyperglycemia [43]. Its synergistic anti-inflammatory and antioxidant effects, limiting oxidative stress in the liver and adipose tissue, contribute to improved insulin sensitivity and metabolic homeostasis [82].

## 5. Formulation, Dosage, and Clinical Context of Zingiberaceae Supplements

In the pharmacological treatment of type 2 diabetes, metformin, sodium-glucose cotransporter 2 (SGLT-2) inhibitors, and glucagon-like peptide 1 (GLP-1) receptor agonists are considered first-line drugs [5]. Key Zingiberaceae species, *Zingiber officinale*, *Curcuma longa*, and *Alpinia galanga*, are increasingly incorporated into dietary interventions to improve glycemic control in type 2 diabetes, as summarized in Table 5. Selecting the appropriate form of administration and optimizing dosage are key elements of effective therapy. It is also worth noting that two or more drugs administered together may cause chemical or pharmacological interactions, which can modify the effects of each drug, resulting in altered efficacy and a changed profile of adverse effects [83]. Previous reviews of herb-drug interactions in diabetes indicate that concomitant use of plant-derived preparations with antidiabetic drugs may lead to pharmacodynamic interactions, particularly additive glucose-lowering effects, and pharmacokinetic modulation of drug metabolism or transport, although most evidence remains indirect and derived from preclinical or heterogeneous clinical studies [83]. It should be emphasized that findings derived from isolated compounds should be interpreted with caution, as they do not fully reflect dietary intake of Zingiberaceae plants. We focus on dose-defined preparations consistent with the definition of dietary supplement interventions used throughout this review.

### 5.1. Zingiber officinale Roscoe (Ginger)

Ginger is most administered as powdered rhizome in capsule or food form. The poor water solubility of natural antioxidants restricts their bioavailability and therapeutic use. The use of phytosomes, liposomes, or nanoemulsions significantly increases the bioavailability, stability, and tissue distribution of gingerols [84,85].

In one study, the bioavailability of four main active compounds in the *Z. officinale* rhizome extract was determined: 6-gingerol, 10-gingerdione, 8-gingerdione, and 8-shogaol. The presence of a food matrix has been shown to reduce the bioavailability of active compounds, with the extent of this effect depending on the type of diet. In the case of each diet, among the studied compounds, 6-gingerol showed the highest bioavailability, followed by 8-gingerdione, 10-gingerdione, and 8-shogaol. The bioavailability of compounds decreased the least in the high-residue diet. It can be concluded that the type of food matrix has a significant impact on the bioavailability of polyphenols present in the ginger rhizome. Also balanced diet promotes their positive interactions with nutrients, which can act as carriers and protectors against oxidative degradation [86].

Clinical doses vary, but the most frequent range is 1–3 g per day, typically for 8–12 weeks [55,87,88,89,90,91]. One study demonstrated the effectiveness of 2 g per day for 12 weeks, resulting in significant reductions in fasting blood glucose and HbA1c [90]. Another trial used a lower dose of 1.2 g per day for 90 days, also reported beneficial changes in body weight and glycemic indices [55]. A systematic meta-analysis showed that doses ranging from 0.5 to 3 g per day administered as capsules for up to 3 months were consistently reported as effective in improving glycemic control [87].

### 5.2. Curcuma longa L. (Turmeric)

Curcumin, the main bioactive component of *C. longa*, exhibits low bioavailability, which limits its therapeutic efficacy [92]. To overcome the low bioavailability of curcumin, several strategies have been developed to enhance its absorption and metabolic stability. Advanced formulations such as co-administration with adjuvants, nanoemulsions, liposomes, encapsulation, nanocarriers, phospholipid complexes, solid lipid nanoparticles, nanogel, and polymeric micelles significantly improve their solubility, intestinal permeability, and systemic circulation. Piperine stands out for its significant potential to improve the systemic bioavailability of curcumin. Consequently, formulations integrating curcumin with piperine have gained attention as a viable approach to overcoming curcumin’s poor bioavailability [93,94].

A daily dose of 1.5 g per day pure curcumin for 12 months significantly improved β-cell function, reduced insulin resistance, and lowered body weight in patients with T2DM [95]. To overcome poor absorption, studies use nano-curcumin, liposomal curcumin, piperine-enhanced formulations, or curcumin-phospholipid complexes [96,97]. For instance, 80 mg nano-curcumin for 12 weeks improved glycemic and metabolic parameters, while curcumin with piperine supplementation for 12 weeks reduced triglycerides, glucose, and CRP. These formulations were well tolerated, with no significant adverse events reported. Another study examining the efficacy of combining piperine with curcumin found that 12 weeks of supplementation significantly reduced fasting triglycerides and glucose levels and marginally reduced CRP levels compared to the placebo group [98]. Daily doses of 500–1500 mg of curcumin have been shown to exert safe and effective anti-inflammatory, antioxidant, and metabolic effects, which confirms its potential in the prevention and treatment of diseases such as metabolic syndrome [99].

### 5.3. Alpinia galanga (Greater Galangal)

Unlike *Z. officinale* and *C. longa*, which are supported by clinical evidence, *A. galanga* remains less studied clinically, reflecting its current preclinical status rather than a basis for nutritional recommendation. The main phenolic compound in *A. galanga* is galangin, the key bioactive constituent of its rhizomes. However, galangin exhibits very low bioavailability due to its poor water solubility and sensitivity to temperature, pH, and light, which limits its bioaccessibility when consumed in traditional extract form [100,101,102]. To overcome these challenges, dual-coated liposomes (chitosan-sodium alginate) were developed, significantly increasing the in vitro bioaccessibility of galangin from 23.87% in the crude extract to 73.65% in the liposomal form. These liposomes also demonstrated high encapsulation efficiency and notable stability under simulated gastrointestinal conditions [103]. Similarly, non-aqueous nanoemulsions of *A. galanga* extract achieved a tenfold increase in skin permeation compared to the extract alone, indicating enhanced topical bioavailability. These nanoemulsions exhibited high stability and efficacy in animal models [39].

Oral administration of methanol extract (200–400 mg/kg body weight) for 21 days in streptozotocin-induced diabetic rats significantly reduced fasting blood glucose and improved lipid profiles [104]. In vitro studies confirm α-amylase and α-glucosidase inhibition, suggesting reduced carbohydrate absorption. Phenylpropanoids isolated from *A. galanga*, especially (1′S)-1-acetoxyeugenol acetate, stimulate GSIS and inhibit α-glucosidase, supporting glycemic control [77]. Despite these encouraging data, there is a notable lack of randomized clinical trials in humans, making it premature to recommend *A. galanga*.

**Table 5:** Studies on the dosages of plants from the Zingiberaceae family.

Plant	Dosage	Study Design	Time	Participants	Effect	Reference
*Zingiber officinale* Roscoe	1200 mg/d ginger powder	Randomized clinical trial	90 days	Type 2 diabetic patients	↓ FBG, ↓ TC, ↓ LDL	[55]
	1800 mg/d ginger powder	Randomized, single blind, placebo-controlled clinical trial	8 weeks	Newly diagnosed type 2 diabetic patients	↓ BMI, ↓ FBG, ↓ HOMA-IR, ↓ HbA1c, ↓ TC, ↓ LDL, ↓ TG, ↓ fasting insulin levels	[88]
	2000 mg/d ginger powder	Randomized double-blinded placebo-controlled clinical trial	3 months	Type 2 diabetic patients with NAFLD	↓ SBP, ↓ DBP, ↓ serum insulin levels, ↓ HOMA-IR	[89]
	2000 mg/d ginger powder	Randomized, double blind, placebo-controlled clinical trial	12 weeks	Type 2 diabetic patients	↓ FBG, ↓ HbA1c, ↓ apoB, ↓ apoA1, ↓ apoB/apoA1, ↓ MDA	[90]
	1197 mg/d ginger powder	Single-arm clinical trial	6 weeks	Type 2 diabetic patients	↓ HbA1c, ↓ TG, ↓ diurnal DBP, ↓ diurnal MAP, ↓ 24 h DBP	[91]
*Curcuma longa* L.	1500 mg/d curcumin	Randomized controlled trial	12 months	Type 2 diabetic patients	↓ FBG, ↓ HbA1c, ↓ HOMA-IR, ↓ leptin, ↑ adiponectin, ↓ BMI	[95]
	500 mg/d of curcuminoids with 5 mg/d of piperine	Double-blind randomized controlled trial	12 weeks	Type 2 diabetic patients with hypertriglyceridemia	↓ FBG, ↓ TG, ↓ TC, ↓ CRP	[98]
	1000 mg/d curcumin with 10 mg/d of piperine	Randomized controlled trial	8 weeks	Type 2 diabetic patients	↑ TAC, ↑ SOD, ↓ MDA	[105]
	80 mg curcumin	Pilot, double-blind, placebo-controlled trial	12 weeks	Older adults with prediabetes or overweight/obesity	↓ HbA1c, ↑ AST levels, ↓ ALT/AST ratio	[106]
	1000 mg/d curcumin	Randomized, double-blind, placebo-controlled trial	12 weeks	Type 2 diabetic patients with coronary heart disease	↓ MDA, ↑ TAC, ↑ GSH, ↓ PSQI	[107]
*Alpinia galanga*	(1′S)-1′-Acetoxyeugenol acetate (AEA)—5 and 10 μM	Controlled in vitro experimental study	24 h	INS-1 pancreatic β-cells	↑ GSIS, ↑ IRS-2/PI3K/Akt pathway protein expressions; ↓ α-glucosidase	[77]
	Methanolic extract of *A. galanga* 200 and 400 mg/kg body weight	Non-randomized, controlled in vivo experimental study	21 days	STZ-induced diabetic rats	↓ FBG, ↓ body weight, ↓ TG, ↓ TC, ↓ LDL, ↑ HDL	[104]

HbA1c—Hemoglobin A1c, DBP—Diastolic Blood Pressure, NAFLD—Non-Alcoholic Fatty Liver Disease, SBP—Systolic Blood Pressure, MAP—Mean Arterial Pressure, HOMA-IR—Homeostasis Model Assessment of Insulin Resistance, BMI—Body Mass Index, FBG—Fasting Blood Glucose, TG—Triglycerides, TC—Total Cholesterol, LDL—Low-Density Lipoprotein, HDL—High-Density Lipoprotein, AST—Aspartate Aminotransferase, ALT—Alanine Aminotransferase, apoB—Apolipoprotein B, apoA1—Apolipoprotein A1, MDA—Malondialdehyde, TAC—Total Antioxidant Capacity, SOD—Superoxide Dismutase, GSH—Glutathione, PSQI—Pittsburgh Sleep Quality Index, GSIS—glucose-stimulated insulin secretion; IRS-2—insulin receptor substrate-2; PI3K—phosphatidylinositol 3-kinase; Akt—protein kinase B, ↓—decrease, ↑—increase.

## 6. Discussion

Although evidence supports antidiabetic and anti-inflammatory activity for Zingiberaceae phytochemicals, translation into nutritional practice requires integrating biological plausibility with intervention type, dosing and clinically meaningful outcomes.

In this review, the term “dietary supplement interventions” refers to targeted, dose-specific nutritional strategies implemented using preparations derived from the Zingiberaceae family with controlled composition and standardized content. These interventions include dried plant preparations, standardized extracts and preparations with increased bioavailability.

The main interpretative limitation of the current literature is that most human studies evaluate Zingiberaceae preparations as supplemental add-ons to standard antidiabetic therapy, rather than as standalone dietary strategies [16,22]. Therefore, claims regarding dietary applicability are often inferred indirectly from supplement-based outcomes. This distinction is critical, as supplementation allows controlled dosing and defined exposure, whereas food-based incorporation introduces substantial variability related to portion size, preparation, digestion, and background diet.

Across studies, the absence of clearly established dose-exposure-outcome relation-ships further constrains interpretation. Even when similar doses are administered, systemic exposure may differ substantially depending on formulation, bioavailability, and background diet. This limitation is particularly relevant for phytochemicals characterized by low and variable bioavailability, where dose alone is an insufficient indicator for biological effect [32]. Therefore, reported dose ranges cannot be used to formulate evidence-based recommendations.

The dietary context may be an additional factor modifying the effectiveness of the supplement. For ginger, evidence from studies indicates that the food matrix can influence phytochemical bioavailability, suggesting that meal composition and timing may alter systemic exposure even in supplementation-based interventions [86]. However, such factors are rarely reported systematically, limiting cross-study comparability and interpretation.

The formulation used determines the significance of dietary supplements. Curcumin, for example, exhibits intrinsically poor bioavailability and is therefore predominantly evaluated using absorption-enhancing delivery systems. As a result, clinical outcomes should be interpreted in relation to the specific formulation used rather than attributed to curcumin as a separate intervention [32,34]. Failure to account for formulation differences risks conflating pharmacokinetic limitations with biological inefficacy.

In contrast, Alpinia galanga remains positioned at an earlier stage. Although preclinical studies demonstrate probability and biological activity, galangin and related compounds show very low bioavailability, and most formulation strategies remain experimental. The absence of well-designed human trials currently precludes evidence-based recommendations even within a dietary supplement framework [38,39].

Another consideration is the frequent co-administration of Zingiberaceae supplements with antidiabetic drugs [83]. While additive or synergistic effects are often implied, few studies are explicitly designed to disentangle independent supplement effects from drug-nutrient interactions. From a dietary supplement intervention perspective, this underscores the need for safety-oriented trial designs incorporating systematic adverse-event reporting and hypoglycemia monitoring.

In summary, the evidence supports a tiered framework for Zingiberaceae species within dietary supplement interventions. *Zingiber officinale* and *Curcuma longa* occupy an intermediate tier, supported by randomized controlled trials and meta-analyses using standardized preparations, yet still limited by short intervention durations and reliance on surrogate endpoints. In contrast, *Alpinia galanga* remains confined to a preclinical tier, where promise exceeds clinical validation. Advancing these plants toward evidence-based nutritional application will require trials explicitly designed to bridge supplement-based efficacy with real-world exposure, formulation transparency, and clinically relevant outcomes.

### Limitations

The available evidence on Zingiberaceae plants is affected by several methodological limitations. Many clinical studies are characterized by small sample sizes, short intervention periods, and substantial heterogeneity in study design, populations, formulations, and outcome measures, which limits direct comparison across studies.

## 7. Conclusions and Future Research

This review critically assesses the evidence for Zingiberaceae-based supplementation interventions for diabetes, distinguishing biological plausibility from clinically effective nutritional use. Although in vitro, in vivo, and human studies support antidiabetic and anti-inflammatory effects of key phytochemicals, current evidence is insufficient to support confident dietary recommendations beyond controlled supplementation settings.

Randomized controlled trials and meta-analyses suggest potential metabolic benefits, particularly for *Zingiber officinale* and *Curcuma longa*. However, reported effects are highly dependent on composition, dosage, and clinical context. In contrast, the evidence for *Alpinia galanga* remains largely preclinical.

In summary, the findings support an evidence-based perspective in which efficacy demonstrated in supplement-based interventions is established before dietary relevance can be considered. Progress in this area will require well-designed randomized controlled trials that clearly assess dose-exposure-response relationships, formulation-specific efficacy, and long-term safety. These efforts are essential to translate controlled supplementation into realistic dietary contexts and to enable evidence-based application of Zingiberaceae-based interventions in metabolic health.

## Figures and Tables

**Table 1:** Diagnosis of diabetes based on laboratory criteria established by international and national guidelines [2,5].

Condition	Diagnosis
Presence of classical symptoms of hyperglycemia and random plasma glucose	≥200 mg/dL
Fasting plasma glucose measured on two separate occasions	≥126 mg/dL
Plasma glucose at 120 min of the 75 g oral glucose tolerance test (OGTT)	≥200 mg/dL
Glycated hemoglobin (HbA1c)	≥6.5%

**Table 3:** Major bioactive constituents and pharmacological activities of *Curcuma longa* L.

Plant	Fraction	Chemical Type	Examples of Main Compounds	Effect	Reference
*Curcuma longa* L.	Volatile	Monoterpenes	p-cymen, 1,8-cineol	Antimutagenic	[26,30]
		Sesquiterpenes	Ar-turmerone, α-turmerone, β-turmerone, curlone	Anti-inflammatory, antimicrobial	[26,30,33,36]
	Non-volatile	Curcuminoids	Curcumin, demethoxycurcumin, bis demethoxycurcumin	Anti-inflammatory, antidiabetic, hepatoprotective, neuroprotective, antimicrobial, anticancer, immunomodulatory	[15,30,37]
		Polyphenols/ flavonoids	Ferulic acid, quercetin, caffeic acid, coumaric acid	Antioxidant, antibacterial	[30,31,37]

## Data Availability

No new data were created or analyzed in this study. Data sharing is not applicable to this article.

## Abbreviations

The following abbreviations are used in this manuscript:

DM	Diabetes mellitus
IRS	Insulin receptor substrate
PI3K	Phosphoinositide 3-kinase
Akt	Protein kinase B
OGTT	Oral glucose tolerance test
HbA1c	Glycated hemoglobin
T1DM	Type 1 diabetes
T2DM	Type 2 diabetes
GDM	Gestational diabetes mellitus
LADA	Latent autoimmune diabetes in adults
GLUT4	Glucose transporter type 4
CUR	Curcumin
DMC	Demethoxycurcumin
BDMC	Bisdemethoxycurcumin
ACA	1′S-1′-acetoxychavicol acetate
AEA	1′S-1′-acetoxy-eugenol acetate
HPLC	High-performance liquid chromatography
GC-MS	Gas chromatography-mass spectrometry
NF-κB	Nuclear factor kappa-light-chain-enhancer of activated B cells
AMPK	AMP-activated protein kinase
CNS	Central nervous system
MAPK	Mitogen-activated protein kinase
FBG	Fasting blood glucose
HOMA-IR	Homeostatic Model Assessment for Insulin Resistance
QUICKI	Quantitative insulin sensitivity check index
PPARγ	Peroxisome proliferator-activated receptor gamma
TNF-α	Tumor necrosis factor α
IL-6	Interleukin-6
IL-1β	Interleukin-1 β
MCP-1	Monocyte chemoattractant protein-1
RANTES	Regulated on activation, normal T-cell expressed and secreted
SOD	Superoxide dismutase
CAT	Catalase
TAC	Total antioxidant capacity
TLR4	Toll-like receptor 4
NLRP3	Nucleotide-binding Leucine-rich repeat and Pyrin domain containing 3
JAK/STAT	Janus Kinase/Signal Transducer and Activator of Transcription
iNOS	Inducible nitric oxide synthase
CRP	C-reactive protein
ROS	Reactive oxygen species
GPx	Glutathione peroxidase
AGEs	Advanced glycation end products
MDA	Malondialdehyde
LDL-C	Low-density lipoprotein cholesterol
VLDL-C	Very low-density lipoprotein cholesterol
HDL-C	High-density lipoprotein cholesterol
GSIS	Glucose-dependent insulin secretion
NO	Nitric oxide
COX-2	Cyclooxygenase-2
GSK-3β	Glycogen synthase kinase-3 beta
SGLT-2	Sodium-glucose cotransporter 2
GLP-1	Glucagon-like peptide 1
